# Intratympanic Dexamethasone Role in Hearing Protection in Cancer Patients

**DOI:** 10.7759/cureus.44299

**Published:** 2023-08-28

**Authors:** Dipti Gupta, Gurbax Singh, Pardeep Garg, Ratul Dey, Baltej Singh

**Affiliations:** 1 Ear, Nose, and Throat (ENT), Guru Gobind Singh Medical College and Hospital (GGSMCH), Faridkot, IND; 2 Otolaryngology - Head and Neck Surgery, Guru Gobind Singh Medical College and Hospital (GGSMCH), Faridkot, IND; 3 Radiotherapy, Guru Gobind Singh Medical College and Hospital (GGSMCH), Faridkot, IND; 4 Audiology, Guru Gobind Singh Medical College and Hospital (GGSMCH), Faridkot, IND; 5 Statistics, Guru Gobind Singh Medical College and Hospital (GGSMCH), Faridkot, IND

**Keywords:** pta, dpoae, cancer, hearing, intratympanic dexamethasone

## Abstract

Introduction

The study aims to determine the role of intratympanic dexamethasone (ITD) on the hearing profile of patients with head and neck cancer post-chemoradiotherapy.

Study design

This study employs a prospective case-control design.

Subjects and methods

In total 834 patients were evaluated for eligibility. Seven hundred and eleven were excluded because they didn’t meet the inclusion criteria. A hundred cases out of 123 were diagnosed with head and neck cancer for which the treatment protocol included cisplatin concurrent to radiotherapy recruited. Before each cisplatin treatment session, ITD was injected into one ear (experimental ear) while the other ear of the same patient served as the control. Pure-tone audiometry (PTA) and distortion product otoacoustic emissions (DPOAE) test results of the baseline and follow-up examinations in the sixth and 12th weeks were compared within and between the study and control ears.

Results

For pure tone thresholds, significant hearing threshold change was noticed at 8 kHz in the experimental group at six weeks and at ≥ 6 kHz in the control group. At 12 weeks, high frequencies were significantly affected at ≥ 4 kHz in the control group. When the baseline was compared across the groups in the 12th week, for otoacoustic emissions, high frequencies showed a loss in the control group more compared to the experimental side (Wilcoxon signed-rank test).

Conclusion

ITD functions less effectively at higher frequencies because the basal turn of the cochlea is more susceptible to cisplatin ototoxicity. ITD might have potential in the reduction of cisplatin-induced hearing loss.

## Introduction

Cisplatin, a most familiar chemotherapeutic agent approved by the US Food and Drug Administration (FDA) in 1978, is used to treat different types of cancers [1]. According to a review of the literature, the incidence of hearing loss caused by cisplatin was around 62% (the range was 11-97%) causing some of the major side effects like ototoxicity, nephrotoxicity, peripheral neurotoxicity, gastrointestinal toxicity, hematological toxicity, etc. [2,3]. With chemotherapy and high radiation dosages, ototoxicity is the most often reported adverse effect [3].

The choice of treatment for these cancers was concurrent chemotherapy with radiotherapy (external beam radiation therapy (EBRT)). EBRT delivers high-energy rays to cancer-specific sites using a specific X-ray machine known as a linear accelerator. In this the machine moves around the patient's body, directing higher and more precise doses of radiation at the cancer-specific site while minimizing damage to healthy tissue and nearby organs. As a result, modern EBRT aids in reducing the risk of side effects associated with traditional radiation therapy [4,5]. Chen et al. (2006) observed radiation doses of greater than 60 Gy in the cochlea were the most significant factor for the presence of sensorineural hearing loss (SNHL) when conventional radiation therapy was used [6].

The mechanism behind ototoxicity postulated is cell death owing to the generation of reactive oxygen species (ROS), which interferes with the antioxidant security system of the organ of Corti and other inner ear structures. This in turn leads to the loss of outer hair cells which chiefly contributes to the hearing loss. If it affects the end organs of hearing, the subsequent effect is diminished hearing sensitivity. A review of the literature revealed that initially the first row, second row, and last third row of the cochlea's outer hair cells are impacted by ototoxic substances. Ototoxicity's primary symptoms are tinnitus, vertigo, and hearing loss. Depending on the underlying pathophysiology, these symptoms may be bilateral or unilateral. It has been observed that there is a positive correlation between cumulative dose of cisplatin and ototoxicity. Hearing loss increases with an increase in cumulative dosage. Factors like cumulative drug dosage, age, physiological, genetic, and biochemical change pose another challenge to monitor ototoxicity-induced hearing changes. Thus, making ototoxicity monitoring a difficult task. If the vestibular system is compromised, there may be symptoms such as nausea and vomiting, imbalanced gait, and dizziness [7]. Overall this results in several social, emotional, physical, occupational, and financial disadvantages for the patient [8]. American Speech and Hearing Association (ASHA) framed guidelines to monitor hearing loss owing to its ototoxicity. It states that an audiologist should obtain a baseline audiogram before starting treatment. ASHA in 1994 stated three main criteria to label ototoxicity-induced hearing loss - (1) evident change in pure tone audiometric threshold of ≥20 dB loss at one test frequency; (2) ≥ 10 dB decrease at any two adjacent test frequencies; or (3) loss of response at three consecutive test frequencies where responses were previously obtained. The third condition stands for the highest frequencies where the earlier subject was able to act in response close to the limits of the audiometer. Moreover, testing should be done in a sound-treated room without any ambient noise presence. Additionally, it stated that ototoxicity-induced hearing loss can be monitored by pure tone audiometry (PTA) and extended high-frequency audiometry testing if possible (ASHA, 1994) [9]. However, being a subjective test, patients can differ in their ability to respond. Additionally, the usage of ultra-high frequency audiometry is also of less importance because of its fatiguing and long-time-consuming nature.

The development of objective measures that do not require patient cooperation is necessary to monitor all patients receiving ototoxic drugs. Otoacoustic emissions are one such objective measure that can be used to detect and monitor ototoxicity-induced hearing changes. Once middle ear functioning is found to be normal, patients can be used to predict hearing-related changes at the cochlear level. Responses are dependent on the physiological status of the outer hair cells which are typically affected first by most ototoxic medications and therefore should be a sensitive and specific measure of hearing change. It has been proposed as an approach for monitoring patients who are not able to respond subjectively or partially. However, there are no accepted protocols for ototoxicity monitoring using otoacoustic emissions, particularly for individuals with preexisting hearing loss.

Intratympanic administration of drugs is a contemporary method of locally treating inner ear disorders, allowing diffusion across the round window into the inner ear where it can exert its effects. Diffusion across the round window into the inner ear and the inner ear structure has been seen because of placing steroids into the middle ear [10,11]. Compared to oral or parenteral routes, this method allows the concentration of steroids to much higher levels within the inner ear [12,13]. Also, to avoid the common systemic side effects of steroids including hyperglycemia, peptic ulcers, hypertension, and osteoporosis, and more problematic, reduced efficacy of chemotherapeutic agents [14], local administration is given which prevents systemic absorption [11,15].

Dexamethasone's ability to reduce cisplatin-induced hearing loss was hypothesized to be due to the cochlea's many mineralocorticoid and glucocorticoid receptors. The spiral ganglia, organ of Corti, spiral ligament, and vestibular sensory epithelium all contain these receptors. The importance of steroids in preserving inner ear homeostasis is demonstrated by the high affinity of mineralocorticoid receptors for steroidal hormones. The blood labyrinth barrier of the inner ear acts as a barrier to prevent dangerous compounds from escaping from the blood after systemic delivery. This blood-labyrinthine barrier deteriorates when ROS are created in excess, reducing the cochlear potentials further, which causes the inner ear's structures to undergo programmed cell death. Glucocorticoids aid in restoring function and maintaining the inner ear's equilibrium [16-19].

To treat inner ear disorders such as Meniere's disease, autoimmune inner ear disease, and cases involving sudden and noise-induced SNHL, medications can be delivered intratympanically [17-19]. Placing steroids in the middle ear caused diffusion over the round window into the inner ear and saturated the inner ear structures. This technique enables steroid concentration to reach much higher levels within the inner ear than oral or parenteral approaches [18,19]. To prevent the common systemic side effects of steroids, such as hyperglycemia, peptic ulcers, hypertension, and osteoporosis, as well as the lessening of the effectiveness of chemotherapeutic drugs, local administration is also employed [20].

There is emerging research that underscores encouraging prospects for the prevention of cisplatin-induced ototoxicity. For instance, research on small mammals has extensively documented the use and efficacy of intratympanic dexamethasone (ITD) [21-23]. The usefulness of ITD at regulated timings in cases of cisplatin-induced ototoxicity has only been studied in a few human studies. There are few studies done to see - the ITD effect on hearing in cisplatin-based chemotherapy cases [20,24]. It is anticipated that the present study will benefit the large population who suffer from cisplatin-induced hearing loss due to ototoxicity in head and neck cancer patients (HNCs).

## Materials and methods

The aim of the current study was to examine the effect of ITD on hearing air conduction (AC) thresholds on subjects receiving concurrent chemotherapy with external beam radiotherapy for HNCs.

The objectives are to evaluate the effect of ITD on audiometric thresholds (AC) (250, 500, 1000, 2000, 4000, 6000, and 8000 Hz). The second objective is to evaluate the effect of ITD on distortion product otoacoustic emissions (DPOAE) signal-to-noise ratio (SNR) at the f2 frequencies from 988 to 8kHz in the first (pre-treatment), sixth, and 12th week and to compare the change in audiometric (AC) thresholds and DPOAE SNRs at pre-treatment with sixth week and 12th week respectively.

The present study was done at a tertiary care teaching hospital. This was a prospective case-control study done in the Department of ENT and the Department of Radiotherapy between January 2017 and January 2022. It aims to determine the effect of ITD administration in preserving the hearing status of individuals who had received concurrent chemoradiotherapy for the treatment of head and neck cancers. The audiological test battery used subjective and objective measures, i.e. PTA and DPOAEs to test the auditory function for ototoxicity. While PTA is a subjective test of auditory functioning, DPOAEs offer a sensitive measure to check the functional conduction of outer hair cells, hence, we selected them as a marker of cochlear function. The objective is to evaluate the effect of ITD on cisplatin-based chemotherapy cases. The present study was done at a 1000-bed tertiary care teaching hospital.

In total 834 patients were evaluated in day care cancer OPD for eligibility. Seven hundred and eleven were excluded because they did not meet the inclusion criteria. The interest of the current study is carcinomas of the HNCs. Later constitutes the oral cavity (n=6 cases), nasopharynx (n=19), oropharynx (n=35), laryngopharynx (n=23), larynx (n=15), nasal cavity (n=2) and paranasal sinuses (none), and major salivary glands cancers (none). A total of 100 cases out of 123 were diagnosed for HNCs for which the treatment protocol included cisplatin concurrent to radiotherapy recruited. Twenty-three patients were lost to follow-up because 15 patients opted to withdraw their consent for participation in the study and in eight cases the treatment protocol was changed by the oncologist before a cumulative dose of 300 mg cisplatin was reached.

Before each cisplatin treatment session, ITD was injected into one ear (experimental ear) while the other ear of the same patient served as the control. The experimental ear side is based on the criteria of the side affected with cancer and the other side acted as a control. Sixty-eight men and 32 women have completed the study. ITD was injected into the right and left ears in 70 and 30 subjects respectively based on the site of cancer. PTA and DPOAEs test results of the baseline and follow-up examinations at the sixth and 12th weeks were compared within and between the study and control ears.

Individuals with diagnosed HNCs and who were planned to undergo concurrent chemotherapy with EBRT as the first line of treatment were included in the treatment. Individuals with pre-existing otological problems like aural fullness, ear discharge, hearing loss, tinnitus, or vertigo and with a history of prior treatment of carcinoma were excluded.

The data was collected from every participant individually in the presence of their attendant, in a quiet sound treated room. Informed written consent was obtained from all the willing patients. A detailed case history was taken before starting of procedure to rule out any ear-related abnormalities. An expert otorhinolaryngologist performed an otoscopy on both ears before performing the audiological evaluation.

The baseline test was PTA and DPOAEs before the start of chemoradiotherapy. Intratympanic injection i.e. 0.5-0.7 mL of dexamethasone (4mg/mL) was injected into the experimental ear an hour before the start of chemoradiation therapy via myringotomy. The otolaryngologist performed the procedure under local anesthesia using cotton wool soaked with Xylocaine 10% which was attached to the tympanic membrane for 15 minutes. Each time 0.5-0.7 mL ITD was injected into the middle ear using a 26-gauge spinal needle via tympanic membrane posterior- inferior quadrant facing the round window niche before each weekly chemotherapy cycle. The patient was instructed to lie down for 30 minutes to avoid swallowing or coughing.

For oncological treatment, the target patient population underwent six cycles of cisplatin chemotherapy majorly and EBRT for #33/66 Gy. Along with concurrent chemotherapy drug cisplatin was given as 500 mL normal saline in a dose of 50 mg at weekly gaps for consecutively six cycles. It was taken care that patients were hydrated with an adequate amount of intravenous fluids i.e. 500 mL dextrose normal saline (DNS) and supplemented with injection KCl, injection MgSO_4_ which was followed by mannitol 200 mL IV, and DNS supplemented with multivitamins Ca and Fe.

Follow-up PTA and DPOAE testing were carried out by any of the three clinical audiologists. Diagnostic 2001 audiometer for recording PTA thresholds. GSI Tympanometer (Grason-Stadler, Inc., USA) to screen the middle ear tympanometry and reflexes, and Neuro-Audio OAEs (Neurosoft, Russia) were used to record OAE thresholds. These were performed first at the pre-start of therapy, second assessment at the sixth week after chemotherapy ended, and third evaluation at the 12th-week post-chemotherapy. At the sixth week, AC thresholds were recorded so as the cumulative dosage of cisplatin reached its maximum, a level at which we expect maximum ototoxic effect after concurrent chemoradiotherapy.

All the participants underwent impedance audiometry (screening) and reflexometry as well to rule out any middle ear pathology.

The probe was inserted in the ear canal using an appropriate size probe tip based on outer ear visual examination of the ear. It was ensured that tests were administered when patients were sitting quietly. The pass criterion for the DPOAEs sound-noise ratio at three consecutive frequencies should be greater than or equal to 6. The frequency range tested was 8 kHz. The study got ethical clearance from the Institutional Ethical Committee vide no. BFUHS/IEC/15/163.

The current study analyzed the audiometric AC thresholds compared between the experimental and control ears. The Saphiro-Wilks test was used to test the normality distribution of data. Descriptive statistics and non-parametric statistical analysis were used to assess the data. Spearman’s correlation of parameters was used to test with the classifiers. To compare the groups, a non-parametric comparison test of mean i.e. Wilcoxon’s Signed Rank Test was used. The parameters were also tested for their relation and the degree of association with independent variables. Appropriate statistics were used for analysis. The data was statistically analyzed using Statistical Package for the Social Sciences (IBM SPSS Statistics for Windows, IBM Corp., Version 21, Armonk, NY).

## Results

During the first pre-chemotherapy phase, 123 patients received ITD in the experimental ear. Fifteen patients chose to withdraw their permission to participate in the study for non-clinical reasons, and the oncologist modified the treatment regimen in eight cases before a cumulative dosage of 300 mg of cisplatin was attained. The study has been completed by 68 men and 32 women. ITD was injected into the right and left ears of 58 and 42 individuals, respectively. The patients' mean age was 58.2+/-6.3 years (males 62.2+/-4.8 years and females 54.1+/-2.6 years).

For all patients, the ITD was given to the experimental ear 60+/-16 minutes before the IV or cisplatin treatment. Except for some dizziness and minor discomfort when administering the medicine, the patients reported relief from ITD injections for tinnitus and hearing in the experimental ear.

Normality of the data

The data was investigated, and it was discovered that the distribution did not match the normal distribution (Shapiro-Wilk test, p<0.05). As a result, non-parametric statistical analysis was used to assess the data.

The data was analyzed using descriptive statistics and non-parametric analysis to identify data-suggested relationships in keeping with the study's goal, which was to determine the efficacy of dexamethasone in avoiding ototoxicity in cisplatin-based treatment in head and neck malignancies. The data was statistically analyzed using IBM SPSS version 21.

The audiometric testing was carried out using the standardized protocol as mentioned in the methodology. The testing was done during three intervals, i.e. pre-treatment, sixth week, and 12th week post the beginning of the concurrent chemoradiotherapy. The correlation of the interval of testing, ear, site, side, and treatment dose of chemotherapy and radiation therapy with the audiometric air conduction threshold is reported in Table 1. The ear grouping based on the laterality of the disease in general was not correlated with any of the audiometric parameters (p>0.05). The side of the ear with ITD (p<0.005, p<0.05) and site of the lesion (p<0.001) correlated with 6000Hz and 8000Hz for AC, and 5714Hz and 8000Hz for DPOAE. Interval of testing (p>0.05) correlated with AC threshold and DPOAEs (p<0.001). The treatment dosage for both chemotherapy and radiation therapy correlated strongly with all the frequencies (p<0.001) mentioned in Table 1.

**Table 1:** Spearman’s correlation of the parameters tested with the classifiers

		Interval	Ear selection	Site of the lesion	Side of the lesion	CT	RT
AC250	Correlation Coefficient	.498^**^	.010	-.022	-.009	.489	.488
	Sig. (2-tailed)	.000	.808	.596	.820	.000	.000
AC500HZ	Correlation Coefficient	.639^**^	.034	.008	-.011	.623	.599
	Sig. (2-tailed)	.000	.408	.845	.785	.000	.000
AC1KHZ	Correlation Coefficient	.026	.050	-.066	.002	.411	.431
	Sig. (2-tailed)	.519	.223	.109	.952	.003	.000
AC2KHZ	Correlation Coefficient	-.441	.020	.048	-.040	-.447	-.394
	Sig. (2-tailed)	.000	.623	.242	.334	.000	.000
AC4KHZ	Correlation Coefficient	.745	.021	-.031	.017	.738	.747
	Sig. (2-tailed)	.000	.607	.450	.686	.000	.000
AC6KHZ	Correlation Coefficient	.846	.000	.200	.127	.831	.821
	Sig. (2-tailed)	.000	.995	.000	.002	.000	.000
AC8KHZ	Correlation Coefficient	.871	-.002	.275	-.099	.855	.829
	Sig. (2-tailed)	.000	.965	.000	.015	.000	.000
OAE988	Correlation Coefficient	-.210	-.004	-.016	.017	-.190	-.191
	Sig. (2-tailed)	.000	.925	.703	.681	.000	.000
OAE1481	Correlation Coefficient	-.381	-.026	.036	-.010	-.364	-.350
	Sig. (2-tailed)	.000	.528	.375	.806	.000	.000
OAE2222	Correlation Coefficient	-.627	.056	-.002	-.019	-.617	-.608
	Sig. (2-tailed)	.000	.172	.961	.645	.000	.000
OAE2963	Correlation Coefficient	-.153	-.023	.035	-.034	-.158	-.148
	Sig. (2-tailed)	.000	.574	.395	.400	.000	.000
OAE4444	Correlation Coefficient	-.345	.003	-.023	-.009	-.352	-.331
	Sig. (2-tailed)	.000	.933	.580	.819	.000	.000
OAE5714	Correlation Coefficient	-.458	-.224	.146	-.048	-.444	-.442
	Sig. (2-tailed)	.000	.000	.000	.243	.000	.000
OAE8000	Correlation Coefficient	-.356	-.220	.115	.009	-.349	-.332
	Sig. (2-tailed)	.000	.000	.005	.833	.000	.000

Experimental and control group

Pretreatment Audiometric Assessment

The PTA (mean air conduction thresholds at different frequencies) of the participants prior to, at six weeks and 12 weeks postchemotherapy is mentioned in Table 2. At baseline, the mean values of AC for the frequencies (in Hz) 250, 500, 1000, 2000, 4000, 6000, and 8000 were comparable in the descriptive statistics. The non-parametric comparison of the mean using the Wilcoxon Signed Rank Test indicated that none of the frequencies showed significant differences (Table 2). At six weeks post the start of chemotherapy when intergroup comparisons (exp. vs. control) were performed, the Wilcoxon Signed Rank Test non-parametric mean comparison revealed that only 6000Hz and 8000Hz demonstrated a significant difference (p <0.05) in accordance with ASHA criteria for ototoxicity (Table 2). At 12 weeks post start of chemotherapy like the second interval of assessment of hearing, 12th week testing yielded distinct patterns across the frequencies while air conduction threshold of 6k Hz and 8k Hz further increased from the levels of sixth week testing. None of the frequencies show significant differences as per ASHA criteria of ototoxicity (Table 2).

**Table 2:** Mean values of AC thresholds for experimental and control groups at three different intervals (baseline, at six weeks, and 12 weeks) AC: air conduction, dBHL: decibels hearing level, Exp: experimental, Cont: control

Average values		Pre (dBHL)		6 week (dBHL)		12 week (dBHL)	
		Exp	Cont	Exp	Cont	Exp	Cont
AC (in Hz)	250	21.55±4.2	22.35±4.8	28.35±4.6	27.6±3.1	27.3±2.5	27.15±2.8
	500	21.25±3.3	21.5±3.5	27.7±4.1	27.45±2.7	27.95±3.2	28.9±3.5
	1	20.15±2.0	20.35±2.8	21.15±2.1	21.3±2.2	19.9±1.6	20.8±2.6
	2	23.95±3.0	23.85±2.9	20.6±1.6	20.3±1.2	21.35±2.2	20.25±2.7
	4	24.75±3.3	24.8±2.8	33.2±3.6	33.7±4.3	35.45±4.6	36.45±4.5
	6	24.6±2.9	24.35±2.5	33.4±3.7	42.65±4.1	42.1±3.2	45.75±3.3
	8	24.45±3.9	24.4±2.7	34.9±4.2	38.6±3.8	41.65±5.6	47.55±3.5

Participants' baseline and sixth-week AC hearing threshold mean values for experimental ears were compared using non-parametric statistical techniques of mean comparison. When compared across the experimental group the difference was not significantly different (ASHA criterion) i.e. hearing thresholds worsen less due to ITD. The difference between the experimental group is 10.45 dB only at 8 kHz (Table 2). The only frequencies where the means change significantly between the control groups (baseline vs. sixth week) according to ASHA criteria for cochlear implant-related hearing loss (CIHL) ototoxicity are at 6kHz (18.3 dB) and 8kHz (14.2 dB), respectively (Table 2).

Comparing the experimental groups' threshold changes at the sixth and twelfth weeks revealed that the experimental groups' threshold changes were 8.7 dB at 6 kHz and 6.75 dB at 8 kHz, while the control group's threshold changes were 3.1 and 8.9 dB at 6 and 8 kHz, respectively. These threshold changes were not clinically significant as per CIHL ototoxicity (Table 2).

The DPOAE values of the participants (experimental and control) before, at six weeks, and at 12 weeks post-chemotherapy are mentioned in Table 3. When baseline DP SNR values were compared for both groups there was no significant change (p<0.05) observed across experimental and control groups (Table 3). After six weeks of treatment, the Wilcoxon Signed Rank Test for DPOAEs revealed a significant difference between the experimental and control groups at 5714 and 8000 (in Hz), i.e., a substantial difference in DPOAEs SNR across groups (Table 3). For DPOAES (SNR cutoff levels <6) decreased from those of the sixth week of testing to 5714 and 8000 Hz (Table 3). According to ASHA's ototoxicity criterion, none of the frequencies exhibit any appreciable variances. It was clinically noteworthy that DPOAEs at 5714 Hz received less than 6 SNR across experimental groups. Significant differences were observed at 5714 and 8kHz between the control groups (p<0.05) (Table 3).

**Table 3:** Mean values of DPOAEs for experimental and control groups at three intervals at baseline, at six weeks, and 12 weeks DPOAEs: distortion product otoacoustic emissions, dBHL: decibels hearing level, Exp: experimental, Cont: control

Average values		Pre		6 week		12 week	
		Exp	Cont	Exp	Cont	Exp	Cont
DPOAE (in Hz)	998	8.0±0.82	7.5±1.66	7.6±2.02	7.7±2.06	7.4±0.64	7.4±1.30
	1481	8.6±0.73	8.1±1.18	7.7±1.80	7.7±1.91	7.4±0.85	7.4±0.75
	2222	8.8±0.83	9.0±1.02	7.6±0.74	8.2±0.68	6.9±0.56	7.8±0.99
	2963	8.7±0.72	8.5±0.84	8.6±0.85	8.3±0.93	8.9±1.13	7.2±0.83
	4444	8.2±0.85	8.1±0.55	8.7±0.84	6.2±0.57	7.9±0.44	6.1±0.90
	5714	7.7±1.30	7.8±1.49	7.6±1.20	5.7±0.93	5.3±0.94	3.4±0.74
	8000	5.5±0.74	5.6±0.69	5.1±0.76	3.9±1.18	5.5±1.36	3.1±0.93

**Figure 1:**
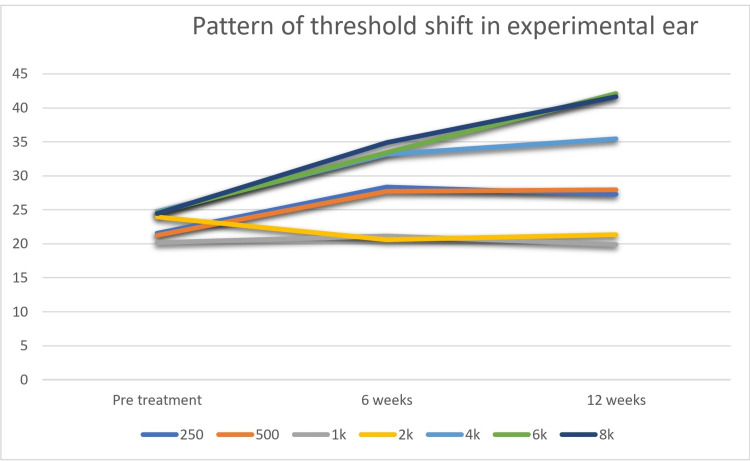
Pattern of threshold shift in the experimental ear (X-axis shows timeline of treatment and Y-axis depicts threshold levels of hearing (dBHL))

**Figure 2:**
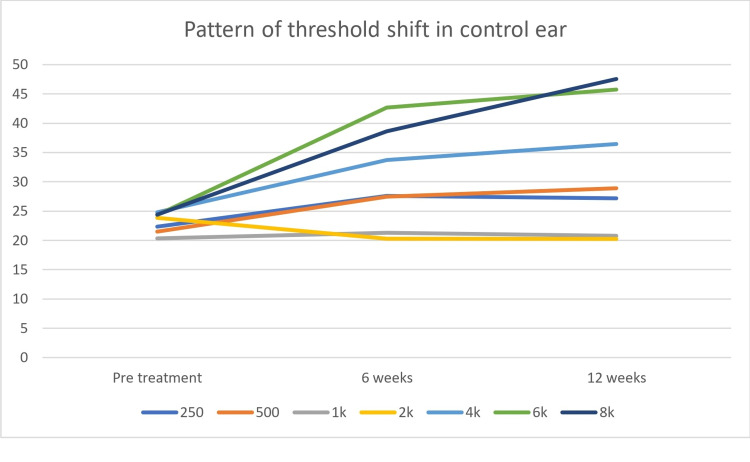
Pattern of threshold shift in control ear

When compared from baseline to the 12th week, a distinct pattern was observed that at high frequencies i.e. 4, 6, and 8 kHz were affected significantly compared to low frequencies in both the groups (Figures 1, 2). When baseline was compared to 12 weeks for control groups using the Wilcoxon Signed Rank Test (non-parametric comparison), a significant reduction in terms of PTA was noticed at high frequencies i.e., 10.7, 17.5, 17.2 dB and 11.65, 21.4, and 23.1dB at 4, 6, and 8 kHz for experimental and control groups respectively (Figures 1, 2).

**Figure 3:**
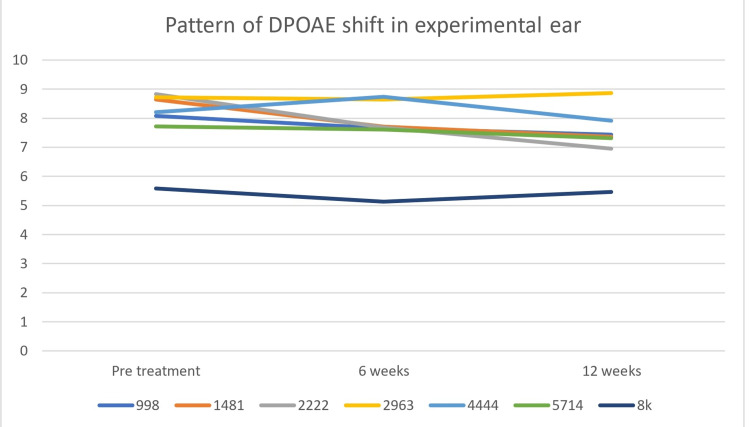
Pattern of DPOAE shift in experimental ear DPOAE: distortion product otoacoustic emission

**Figure 4:**
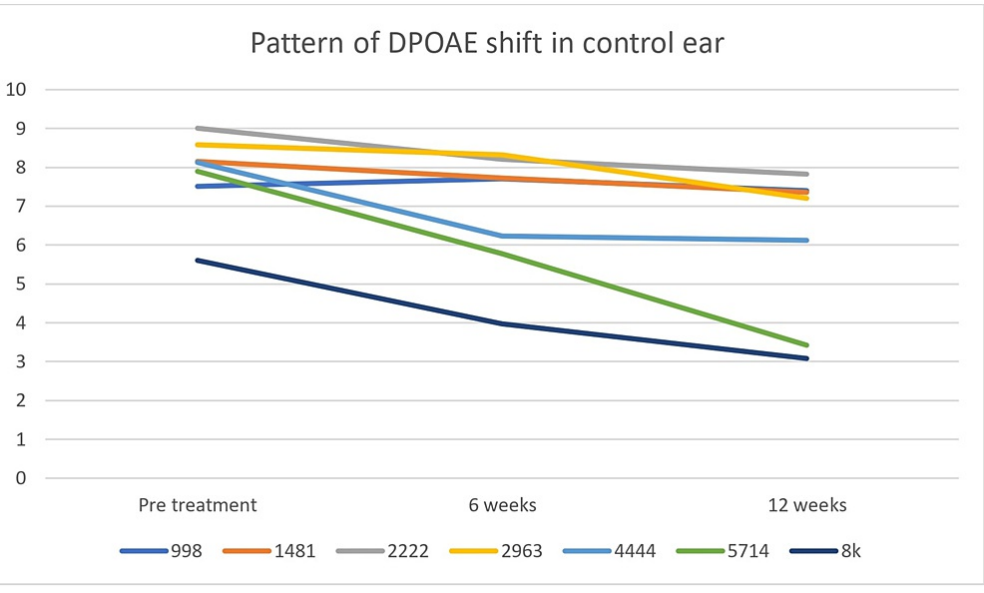
Pattern of DPOAE shift in control ear DPOAE: distortion product otoacoustic emission

Patterns of SNR shift for both lower and higher frequencies were demonstrated in Figure 3 (experimental group) and Figure 4 (control group). The shift of threshold was observed in the lower (998 and 1481Hz) and higher frequencies (4444, 5714, and 8kHz). However, the degree of shift of SNR was greater in the control ear for higher frequencies and the degree was comparable for lower frequencies for both the groups in both DPOAEs (Figures 3, 4). The experimental group and control groups have reduced DPOAEs SNR at 5714 and 8000 Hz only while none show a marked change for the experimental group (Figures 3,4).

Site of Head and Neck Cancers

The audiometric parameters of high frequency (6000 and 8000 Hz for air conduction (Table 4) and 5714 and 8000 Hz for DPOAE SNR values (Table 5)) were significantly related to the site of lesion. Based on the descriptive analysis, the threshold shift was highest for high frequency for the nasopharynx and oropharynx when compared to other sites.

**Table 4:** Chi-square analysis of the site of lesion with AC threshold shift AC: air conduction

AC	250	500	1K	2K	4K	6K	8K
Chi-Square	.489	1.720	5.521	2.478	2.476	130.573	143.034
df	4	4	4	4	4	4	4
Asymp. Sig.	.975	.787	.238	.649	.649	.000	.000

**Table 5:** Chi-square analysis of the site of lesion with DPOAE shift DPOAE: distortion product otoacoustic emission

DPOAE	988	1481	2222	2963	4444	5714	8000
Chi-Square	.489	1.720	5.521	2.478	2.476	130.573	143.034
df	4	4	4	4	4	4	4
Asymp. Sig.	.975	.787	.238	.649	.649	.000	.000

## Discussion

The current study explored the efficacy of ITD injection to prevent ototoxicity during cisplatin-based chemotherapy in HNCs undergoing concurrent chemoradiation therapy.

A total of hundred individuals (n=200 ears) participated in the study with random assignment of ears to the groups, experimental and control groups. All the participants underwent a pre-treatment evaluation using PTA and DPOAEs before the chemotherapy. The ears (participants one ear is assigned to the experimental and another to the control group) in the experimental group received ITD prior to every chemotherapy cycle. All the participants underwent a second interval assessment after six weeks of chemotherapy. Some had completed their chemotherapy while some had a pending cycle. Finally, a third interval assessment was carried out post-12 weeks of the start of chemotherapy treatment.

The audiometric parameters which were AC (250, 500, 1000, 2000, 4000, 6000, 8000 Hz) and DPOAEs SNR frequencies were analyzed within the experimental vs. control group, site of lesion in HNCs (oral cavity, oropharynx, nasopharynx, hypopharynx, and larynx), side of the lesion, and dose of chemotherapy. Hearing loss manifested as SNHL bilaterally that began at high frequencies and gradually extends to speech perception frequencies with increasing dose or extended treatment. This could be due to outer hair cells at the base of the cochlea are said to be impacted by cisplatin first, advancing to apical cells. This finding aligns with the observation reported by Cardinal [21].

The other main finding when compared across the baseline with six-week intervals, the low and mid frequencies do not show a significant change while a significant change (p<0.05) was seen for 8 kHz in the experimental group and for 6 kHz and 8 kHz in control groups. In our study, it was attempted that the dexamethasone was given approximately one hour prior to every chemotherapy cycle. This is due to the antioxidation property of dexamethasone which reduces the formation of the oxygen and nitrogen compounds which then lead to apoptosis and cell damage in the cochlear level. According to researchers, the maximum concentration of cisplatin in the perilymph was detected 20 minutes after IV administration, and it decreased to nearly 50% in the next 40 minutes [22]. Dexamethasone concentration peaked an hour after it was administered and declined to 50-100 times after 12 hours of treatment [12,22]. Interestingly, it shows the role of dexamethasone as an otoprotective agent if given in multiple doses before every chemotherapy cycle [23]. Other frequencies were also relatively less shifted across the interval of testing.

Furthermore, when compared across the sixth- and 12th-week interval, the difference doesn’t show much change. Although loss increased at 4, 6, and 8 kHz in both experimental and control groups, but control group showed more thresholds worsening. With the increase in the dose of cisplatin and increase in time, the threshold shift and amplitude reduction became obvious but loss was not increasing in a significant manner. Other frequencies were relatively less shifted across the interval of testing. The study done by Murphy and Daniel (2011) in animals (12 mg/kg) shows better protection when higher doses were used intratympanically [24]. On the contrary, the study done by another author stated long-term ITD did not prevent CIHL, which employed large doses for lengthy periods of time (8 mg/24 hrs) in the same cases [25].

For the 12th week AC thresholds of 250 and 500Hz became slightly better in contrast with the sixth-week interval testing, while AC thresholds of 6kHz and 8kHz further increased from the levels of sixth week testing. The experimental ears with ITD administration showed reduced threshold shifts in PTA and DPOAEs amplitude reduction across all the frequencies. Our findings correlated with other studies done in small mammals [26,27]. There are very few studies that looked at ITD in the treatment of cisplatin-induced hearing loss cases in human beings [25]. According to Marshek et al. (2014), there was a significant increase in pure tone thresholds at 8000 Hz. The pure tone average at 4000 Hz to 8000 Hz significantly increased in both groups. The control group had a considerable increase in pure tone thresholds for 6000 Hz compared to the study group [20].

The other main finding is when the experimental group was compared across the baseline with sixth- and 12th-week intervals, the low and mid frequencies did not show a significant change while a significant change (p<0.05) was seen for high frequencies. Interestingly, the mid and high frequencies till 6kHz were found to have DP SNR above 6 showing the role of dexamethasone as an otoprotective agent if given in multiple doses before every chemotherapy cycle [23]. This finding is also in corroboration to Marshek’s study where he has also observed reduced DPOAEs SNR for the f2 frequencies 7031 and 8391 Hz [20]. Additionally, research done on guinea pigs also found no change in DPOAEs in the ITD group but a significant decrement in the non-ITD group [26]. On the contrary, this does not happen for the control group where 5714 and 8000 Hz have SNR below 6.

The results of the study indicated that the hearing threshold shift and DPOAE amplitude reduction were seen for higher frequencies and were related to the oropharynx and nasopharynx sites of HNCs. Interestingly, the experimental ear group showed a lower threshold shift for high frequencies than that of the control indicating that ITD administration to the experimental ear may be a safe, simple, and effective intervention that minimizes cisplatin ototoxicity without interfering with the chemotherapeutic actions of cisplatin.

The limitations of the present study are it's possible that the outcomes of the cisplatin test could be misleading due to it being given primarily to an older demographic. It's important to conduct deliberate screenings among younger individuals to ensure accuracy. Additionally, it is challenging to enforce time limitations in clinical settings since patients prioritize the treatment of their fatal diseases.

Clinical implications

Though results of the study indicate that, further exploration may be necessary to conclude on the efficacy of ITD administration, the preventive methods using dexamethasone is necessary to be considered as a part of treatment protocol to reduce the toxicity due to the use of cisplatin for chemotherapy in oncology. Such efforts will reduce the treatment-related symptoms and not cause further burdens on the patient and family members in activities of daily living.

Future directions

It is difficult to consider all the variables and create a homogenous group for a comparative study in oncology. In this study, the efficacy of dexamethasone was evaluated using the threshold shift of AC threshold. A correlation analysis of the site of the HNC indicated that the eustachian tube dysfunction and conductive component of hearing loss may interact with the overall hearing of the person. Such effects need to be studied further to understand such a phenomenon.

## Conclusions

The current study looked at the efficacy of ITD to prevent ototoxicity in patients undergoing chemotherapy in HNCs. The audiometric AC threshold was compared in three intervals: pre-treatment, six, and 12 weeks after the start of the treatment. The results were also correlated with the treatment efficacy within the site and laterality variable.

The results of the study indicated that the hearing threshold shift was seen for lower frequencies and was related to the oropharynx and nasopharynx sites of HNCs. These shifts were noted for both the experimental and control ear groups. Interestingly, the experimental ear group showed a lower threshold for high frequencies than that of the control indicating that ITD administration to the experimental ear may have had a toxicity preventive role.

The shift in low frequencies was between the pre-treatment and six-week post-start of the treatment. However, the difference in low-frequency shifts did not replicate itself between six and 12 weeks after the start of the chemotherapy. Further stringent methodological measures may be necessary to avoid the interaction of variables.

## Figures and Tables

**Figure 5:**
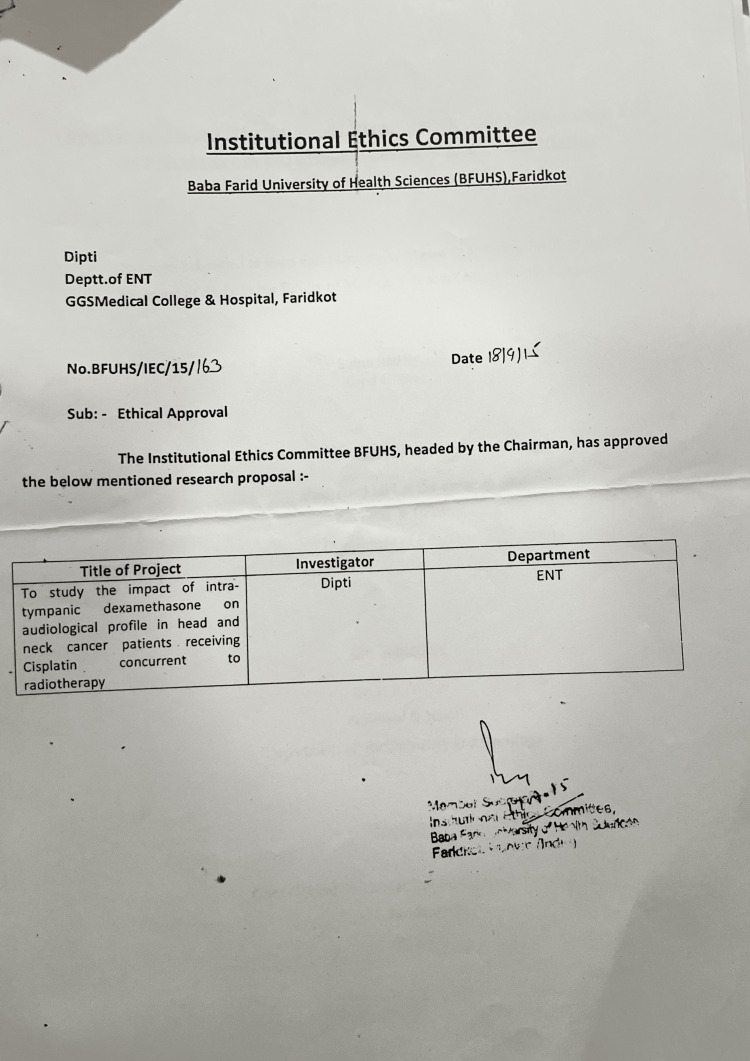
Ethics letter of Institutional Review Board
